# Physiological Action of Nitrous Oxide Gas

**Published:** 1887-06

**Authors:** 


### Physiological Action of Nitrous Oxide Gas.

Dr. Dudley Buxton has communicated two valuable papers upon the above subject to the Odontological Society, based upon numerous clinical observations and experiments. The effects of nitrous oxide inhalations upon the mammalian organisms are, he says broadly speaking—1, a condition of anæsthesia; 2, an emotional state, provoking a sensation of exhileration—in fact, it plays the *role* of stimulant; 3, it gives rise to modifications of the respiratory and 4, circulatory systems; and 5, provokes marked muscular movements which may be classed as (*a*) rigidity and (*b*) jactitations. The anæesthesia produced by nitrous oxide is not dependant upon analgesial or loss of sensation of painful impressions of the sensory end-organs, such as that produced by cocaine, etc., or upon failure of the conducting sensory nerves, for sensation is retained until the perceptive powers themselves cease to receive; moreover, there is immediately anterior to the loss of consciousness a hyperæsthetic stage, therefore it may be concluded that the nerve centers are acted upon. The way by which nitrous oxide may enter the system, and is enabled to produce its special effects are—either that it gives rise to other bodies by changes in its chemical form, or by acting as an irrespirable gas and causing asphyxia, or by exercising a specific action, just as strychnine does. Dr. Frankland came to the conclusion that nitrous oxide was not decomposed during its sojourn in the body, basing his opinion upon analyses made of the air expired by rabbits when confined in an atmosphere of mixed air and nitrous oxide. In the first stage of asphyxia, that of dysnœa there is an increase in the respiratory movements, both inspiratory and expiratory; in the second of a dominance of the expiratory efforts, culminating in general convulsions, in the last, exhaustion, with long-drawn inspiration, gradually dying out. The blood-pressure during the first and second stages rapidly rises. Dr. Dudley Buxton has never observed an increase in the expiratory movements when $HO_2$ has been administered, which are merely increased in number and depth, or expiratory convulsions, notwithstanding the gas has been pushed to its utmost limit, and from a large number of sphygmographic tracing the tension in the arteries has been lower than normal. In experiments upon dogs, Dr. Buxton found that where a trephine-hole was made through the skull, during the inhalation of the gas the brain pulsations became more forcible and somewhat hurried; then brain substance was seen to swell up, until at last it actually protruded through the aperture; whereas in a similar experiment, with the trachea occluded, the brain receded, sinking away from the opening. Other experiments showed that the heart's action was but little interfered with by nitrous oxide, even when inhalations were pushed until respiration was interrupted; during asphyxia, on the other hand, a rapid and continuous increase in blood pressure invariably occurred. The dose of nitrous oxide required to produce insensibility varies very considerably in different persons—a fact which supports the view that nitrous oxide exerts a specific action on the nerve centers. Dr. Buxton also discusses many other interesting points in the action of the gas, such as the occurrence of hallucinations —*Lancet*, April 9, 1887.

